# Spatio-temporal and transmission dynamics of sarcoptic mange in an endangered New World kit fox

**DOI:** 10.1371/journal.pone.0280283

**Published:** 2023-02-16

**Authors:** Patrick Foley, Janet Foley, Jaime Rudd, Deana Clifford, Tory Westall, Brian Cypher

**Affiliations:** 1 Department of Biological Sciences, Sacramento State University, Sacramento, California, United States of America; 2 School of Veterinary Medicine, Department of Medicine and Epidemiology, University of California, Davis, Davis, California, United States of America; 3 Wildlife Health Laboratory, California Department of Fish and Wildlife, Rancho Cordova, California, United States of America; 4 Endangered Species Recovery Program, California State University, Stanislaus, Turlock, California, United States of America; Universitat Autònoma de Barcelona, SPAIN

## Abstract

Sarcoptic mange poses a serious conservation threat to endangered San Joaquin kit foxes (*Vulpes macrotis mutica*). After first appearing in Bakersfield, California in spring 2013, mange reduced the kit fox population approximately 50% until the epidemic ended with minimally detectable endemic cases after 2020. Mange is lethal and thus, with such a high force of infection and lack of immunity, it remains unclear why the epidemic did not burn itself out rapidly and how it persisted so long. Here we explored spatio-temporal patterns of the epidemic, analyzed historical movement data, and created a compartment metapopulation model (named “**metaseir”)** to evaluate whether movement of foxes among patches and spatial heterogeneity would reproduce the eight years epidemic with 50% population reduction observed in Bakersfield. Our main findings from **metaseir** were that: 1) a simple metapopulation model can capture the Bakersfield-like disease epidemic dynamics even when there is no environmental reservoir or external spillover host, 2) the most impactful parameter on persistence and magnitude of the epidemic is the projection, β/α_β_ (transmission over decay rate of transmission over space), 3) heterogeneity in patch carrying capacities changes the critical value of the projection needed to achieve an epidemic but makes little difference to epidemic persistence time, and 4) the epidemic is relatively insensitive to birth rates and density vs. frequency-dependent transmission. Our model can help guide management and assessment of metapopulation viability of this vulpid subspecies, while the exploratory data analysis and model will also be valuable to understand mange in other, particularly den-occupying, species.

## Introduction

Sarcoptic mange (hereafter, mange), the skin disease caused by infestation with the mite *Sarcoptes scabiei*, occurs worldwide in over 100 mammalian species, with a clinical spectrum from mild patchy lesions to loss of integrity and function of the skin, leading to death [1]. With its high case fatality rate in some particularly vulnerable species, mange has the potential to cause significant population decline as has occurred in Northern chamois (*Rupicapra rupicapra*), Iberian ibex (*Capra pyrenaica*), red foxes (*Vulpes vulpes*), coyotes (*Canis latrans*), and wombats (*Vombatus ursinus*) [2–10]. A recent review of sarcoptic mange among wildlife identified outbreaks with ongoing geographic spread, increased virulence, and more host species involved [11], and the disease is considered a global welfare issue and priority conservation concern ([12] and references within).

A sarcoptic mange epidemic poses a serious conservation threat to endangered San Joaquin kit foxes (*Vulpes macrotis mutica*) after first appearing in an urban population in Bakersfield, California in spring 2013, growing to encompass the entire city, with a few cases even eventually detected in the small town of Taft, 56 km southwest of the city, and reducing the overall kit fox population approximately 50% until case numbers abruptly plummeted in 2020 [13] (Cypher pers. comm). In fox family members vulpids such as the San Joaquin kit fox, mange exhibits relatively uniformly rapid, fatal disease progression and there is no evidence for asymptomatic carriers [13].

In a population of highly and homogeneously susceptible and interacting individuals, the rapid disease progression and high lethality of mange would tend to cause an epidemic to flare intensely and then burn out, in part because of a paucity over time of naïve, in-contact individuals that are available to become infected. In addition, if an immune class does develop, this can promote epidemic persistence either if immunity wanes or as new susceptible individuals immigrate or are born. Prolonged mange epidemics in kit foxes have been hypothesized to involve transmission in dens which are used by multiple host species and kit fox families [14]. However, while fox to fox transmission undoubtedly occurs in dens especially among kit fox family groups, mange mites only remain infective off-host for at most a few days [15, 16], so it is unknown if vacant dens are a source of many cases, in contrast with the clear role played by dens in mange in the far less social wombats and bears [12, 17].

Rescue of a faltering mange epidemic from an infested sympatric host species is also unlikely: genetic data suggest there may have been rare spillover from coyotes or dogs and that coyotes may have been the original source for the epidemic in Bakersfield, but that the kit fox epidemic is now maintained by kit fox to kit fox spread of a distinct kit fox-associated mite genotype [18], which is found in no other sympatric host species except red foxes. Ruling out these other mechanisms to maintain the epidemic leaves some form of heterogeneity serving to reduce the force of infestation as a plausible mechanism underlying the observed dynamics. Sources of heterogeneity can be immunological, if some individuals experience prolonged non-lethal infestation (and immunity), or spatial, if the population is divided into smaller, variously interacting subpopulations.

For kit foxes, there does not appear to be another source from which the mite regularly spills over, there is not an immune class, and extensive den transmission could increase the force of infection, possibly increasing the speed with which the epidemic proceeds and then ends. The question of how epidemics can persist in the face of high epidemic amplitude and mortality has been addressed for plague in prairie dogs (*Cynomys ludovicianus*) in the western US, with some researchers suggesting that plague persists in a metapopulation of prairie dog towns [19]. Modeling of plague in California implicated seasonal heterogeneity in flea species on differing small mammal species as the means to maintain the pathogen, *Yersinia pestis*, in nature [20]. Thus we consider whether spatial heterogeneity, defined as an uneven distribution of foxes over space, could be a source of reduced amplitude and prolonged duration of the San Joaquin kit fox mange epidemic.

In this study, we explored spatio-temporal patterns of sarcoptic mange in San Joaquin kit foxes, asking whether spatial heterogeneity in patch carrying capacity, combined with observed kit fox movement, would suffice to maintain the observed disease dynamics even without an environmental reservoir or spillover host. We analyzed historical movement data collected on San Joaquin kit foxes in Bakersfield. We also created a compartment, metapopulation model for mange in this species, aiming to reproduce the persistent 50% reduction in kit fox population size and the rapid onset and abrupt cessation of epidemic case levels after about seven years. We also considered impacts on the model of changing host birth rates (which could infuse varying numbers of juvenile susceptible individuals into a population) and density vs. frequency-dependent disease (DDT vs FDT) transmission. We propose that this model will provide guidance for parasite management and offer some assessment of the population and metapopulation viability of this vulpid subspecies. The exploratory data analysis and model will also be valuable to understand mange in other species.

## Materials and methods

### Natural history of mange in San Joaquin kit foxes

Natural history and disease data were used where possible from the Bakersfield population of San Joaquin kit foxes, and then, in descending order of preference: from San Joaquin kit foxes outside of Bakersfield, from other vulpids, and from other non-vulpids infested with mange. The city of Bakersfield (35.3733 N, -119.0187W, 123 m above sea level) has a large footprint of 390 km^2^ at the far south of the San Joaquin Valley of California and a population of 400,000 people. The area receives only 165 mm/year precipitation as rain, predominantly in winter (www.usclimatedata.com). Summer high temperatures commonly exceed 33.3 °C while minimum temperatures in winter are approx. -1.1 °C. Landscapes surrounding the city have been extensively modified for natural gas and oil extraction, manufacturing, and agriculture including large dairies and grapes, citrus, almonds, carrots, alfalfa, cotton, and roses [20].

The city encompasses large arteries for vehicular traffic including California State Route (SR) 99 running north to south across the city, SR 58 east from SR 99 in the southern part of the city, SR 178 from downtown to the northeast, and the Alfred Harrell Highway along the northern edge of the city to Hart Park in northeast Bakersfield. Constructed areas of Bakersfield include residential subdivisions, large tracts of industrial development, and a sprawling urban core. Open space includes the highly irrigated California State University Bakersfield (CSUB) campus (152 ha), Hart Park on the northeastern edge (150 ha), five golf courses, Lake Ming (201 ha), and the Kern River Parkway (460 ha). While the Kern River does flow into Bakersfield from the Sierra Nevada Mountains to the east, all of its water is channelized into irrigation and aquaducts before entering the city, and all water flow in the city is through concrete and rock-constrained man-made ditches and canals.

San Joaquin kit foxes are charismatic small carnivores endemic to the San Joaquin Valley, obligately using subterranean dens to raise pups and avoid predators and extreme daytime temperatures [21]. There are three natural populations that are considered large or “core” (at Carrizo Plain, Lokern Natural Area, and Panoche Hills) and twelve smaller “satellite” populations, including urban satellites in Bakersfield, Taft, and Coalinga [21–23]. Prior to the beginning of the mange epidemic in 2013, there were approx. 400 kit foxes living in Bakersfield (Cypher, pers. comm.). The San Joaquin kit fox density at the Naval Petroleum Reserve was estimated to be 0.82 foxes/km^2^ [24], while an average estimate across seven sites in Bakersfield, where marked-recapture analysis was applied to individual foxes that were uniquely identified by genotype in scat, was 2.0 kit foxes/km^2^ (range 0.1–4.5) [25]. Kit foxes use an average of 11 dens/year to a maximum of 49/year [21, 26, 27] and the average home range size of a kit fox in Bakersfield is 1.72 km^2^ (± 0.19, 80% overlap among family members, male 2.12 ± 0.31, female 1.36 ± 0.21) [28], yielding an estimate of 6.4 dens/km^2^. Of 471 known occupied dens [29], density is 4.3 dens/km^2^, with the densest cluster of dens on the CSUB campus.

As for many carnivores, movement of kit foxes varies by sex, age, and season, but a thorough analysis of movement in Bakersfield has not been conducted. At the Naval Petroleum Reserves, 32.5% of kit foxes dispersed (defined as long-distance movement outside parental home range), comprising 44% of males and 21% of females [30]. On average, they disperse as subadults at eight months of age, with a range from 4–32 months [30]; depending on the study, dispersal is reported to occur in year one of age from Jun—Dec [21], Jun–Oct [28, 30] or 1 May–Sept 31 [31]. Delayed dispersal is more likely when food availability (and habitat saturation) is high, which are conditions that are typical in Bakersfield [21]. Their movement speed has been estimated at 0.60 km/hour minimum straight-line distance [29]. The mean dispersal distance was 7.8 km [30] although movements > 8 km have been recorded [21]. Interactions among kit fox individuals are extensive within family-groups and quite rare between families: as distinct home ranges are maintained by scent marking, agonistic direct interactions tend to occur between males and from Aug 23-Oct 12 and Feb 16 –Mar 8 [32].

San Joaquin kit foxes breed once per year. They pair-bond from about Nov-Dec, mate in Dec-Jan, and give birth in late Jan- early Mar, with 78.8% reproductive success each year in Bakersfield and an average of 3.8 pups per litter [21, 24, 26, 33]. They typically mate for life, but extra-pair copulation occurs routinely and frequently [21, 32, 34, 35]. Pups are weaned from Mar-May with some parental care into Jun [21]. After pup recruitment, approximately 50–80% of the population comprises adult kit foxes and 20–50% is juvenile [24]. Adult survival is slightly higher among urban compared to exurban kit foxes at 0.7 (range 0.48–0.95) annual survival probability, whereas exurban juvenile annual survival probability is 0.41–0.21, a rate that is likely similar in urban kit foxes [21, 24, 28, 30, 36, 37]. Rates of death are also comparable for dispersers and non-dispersers [30]. On the Naval Petroleum Reserves, death was often due to predation, especially occurring May-Aug for non-dispersing foxes (which may have increased vulnerability to predators if they are inexperienced) and Jun–Oct for dispersers [30]. In Bakersfield, primary causes of death were vehicular strike (41.1%– 53.2%), with far fewer deaths due to predation, poison, and other causes [29]. Male deaths due to cars were more pronounced in Nov–Jan while female deaths were more pronounced in May–Sept.

Kit fox sociality, den use, and relatively high densities within the city of Bakersfield may facilitate the spread of mange mites. In vulpids, mange may initially be subclinical but minimally infectious for approximately three-four weeks (a latent period), after which the mites are transmissable and mange becomes clinically evident [5, 38]. Disease progresses until it terminates with death in about 100 days; individuals do not become immune or survive [1, 5, 12, 38, 39]. Thus compartment models for mange in this species fit well in the susceptible (S), exposed (E), infectious (I) and recovered (R) or SEIR paradigm.

### Kit fox movement

We collated data from 1997–2020 on movement of kit foxes that were monitored with very high frequency (VHF) and satellite telemetry and trapping as part of various other research and conservation projects. Kit foxes were captured using wire-mesh live-traps (38 x 38 x 107 cm, Tomahawk, Hazelhurst, WI) baited with canned cat food, sardines, or hard-boiled eggs and covered with tarps to provide protection from inclement weather and sun. Captured kit foxes were coaxed from traps into a denim bag and handled without chemical restraint. Data collected for each kit fox included date, location, sex, age class (adult or juvenile), mass, overall physical condition, and dental condition. A uniquely numbered metal tag was attached to one ear. Work was conducted in accordance with 10(a)1(A) research permit TE825573-2 from US Fish and Wildlife Service, a Memorandum of Understanding from California Department of Fish and Wildlife (CDFW), and University of California Davis Institutional Animal Care and Use Protocol #18179.

We recorded distance moved as the longest linear distance between any two location points for any kit fox. We summarized the data into the following categories separately by sex and age class: number of kit foxes known to move < 1.5 km, 1.5–3.0 km, 3.0–4.5 km etc., chosen because 1.7 km^2^ is the expected home range of a fox [28] and the diameter of a circular home range of that area would be 1.48 km. We evaluated whether males or females were over-represented in the long-distance movement category with a Fisher’s exact test, grouping “local movement” as anything less than 3.0 km and long-distance as > 3.0 km. Similarly, a Fisher’s exact test was used to evaluate whether age class impacted distance traveled. All statistical analyses were conducted in R [40] and a p-value of ≤ 0.05 was used to infer significance.

### Spatial analysis of kit fox mange case data

Geolocations were obtained for every kit fox found with mange during years 2013–2020. Cases included those that were reported by members of the public or included in various research studies as long as they were verified by authors. Cases were diagnosed as described previously based on consistent lesions which, for kit foxes with advanced sarcoptic mange, are very characteristic [12]. Most cases were also confirmed by microscopic visualization of the mite on skin scrapings. Locations were visualized and analyzed with qGIS (QGIS Geographic Information System, Open Source Geospatial Foundation Project, http://qgis.org). A separate map was created for each year. Maps utilized the “Map data © OpenStreetMap contributors, Map layer by Esri (Redlands CA)”.

Early in the epidemic, there appeared a small number of relatively discrete patches of cases (defined as a “patch”); using the qGIS linear distance measuring tool, we measured the distances to the center of new patches from the center of the most proximate established patch. Because there were multiple cases across several months in each year and within each patch, we used the median month of a year when that patch had reported cases. From the distance moved and difference across each two years in median month of reported cases, we calculated a possible “diffusion rate” as the distance/time before a new patch was observed. The overall diffusion rate was the mean of all measured rates.

### An SEIR model of mange in San Joaquin kit foxes

We modeled mange in San Joaquin kit foxes using a computer simulation (**metaseir** and accessory functions) in R. All methods and R code are open-source and available for use by other researchers and species managers. **metaseir** is a discrete-time Monte Carlo simulation of dynamics in a metapopulation of h patches, and returns the simulated history of the host metapopulation, with total population N adult females divided into S (susceptible), E (exposed), I (infected), and R (recovered) compartments.

SEIR dynamics can be represented by the system of differential equations:

N=S+E+I+R

$$\frac{dS}{dt}=bN-\beta SI+\nu R-d_{S}S$$

$$\frac{dE}{dt}=\beta SI-\sigma E-d_{E}E$$

$$\frac{dI}{dt}=\sigma E-\gamma I\;-d_{I}I$$

$$\frac{dR}{dt}=\gamma I-\nu R-d_{R}R$$

where b is the daily birth rate (allowed to vary with season and local population density); β is transmission rate; ν is the rate at which disease resistance wanes; the d’s are death rates for each compartment, which we assume to be the same for all except the I category; σ is the rate of change from the E state to the I state; and γ is the natural recovery rate which for kit foxes appears to be zero.

Each run of the **metaseir** Monte Carlo simulation produces unique results due to the incorporation of demographic stochasticity. Simulations were run up to tmax days (a trial), archiving a history at 10-day increments of numbers of N, S, E, I, and R individuals; results from multiple trials were tabulated using the function **seirshell**. **metaseir** updates numbers of foxes in each compartment each day using discrete random number generators rpois() and rbinom(), which implements demographic stochasticity for birth-death and SEIR processes, respectively. For generality, the program can allow b to include environmental stochasticity by setting the parameter v_r_ > 0 although this was not done in the present study, i.e. v_r_ is a Normal random variable with expected value = 0 for this study. The birth rate is seasonal with births taking place over the course of 45 days in late winter; we treated death rates as constant across seasons. Density dependence was implemented with a soft ceiling at K, i.e. the population experienced a maximum birth rate (b_m_) at all times when below K, births ceased at K, and the population could occasionally exceed K due to demographic stochasticity. We tracked females, which is an index for families, assuming that once one member of a kit fox household contracts mange, the others quickly do so also (and thus members within a family are not independent).

**Fig 1:**
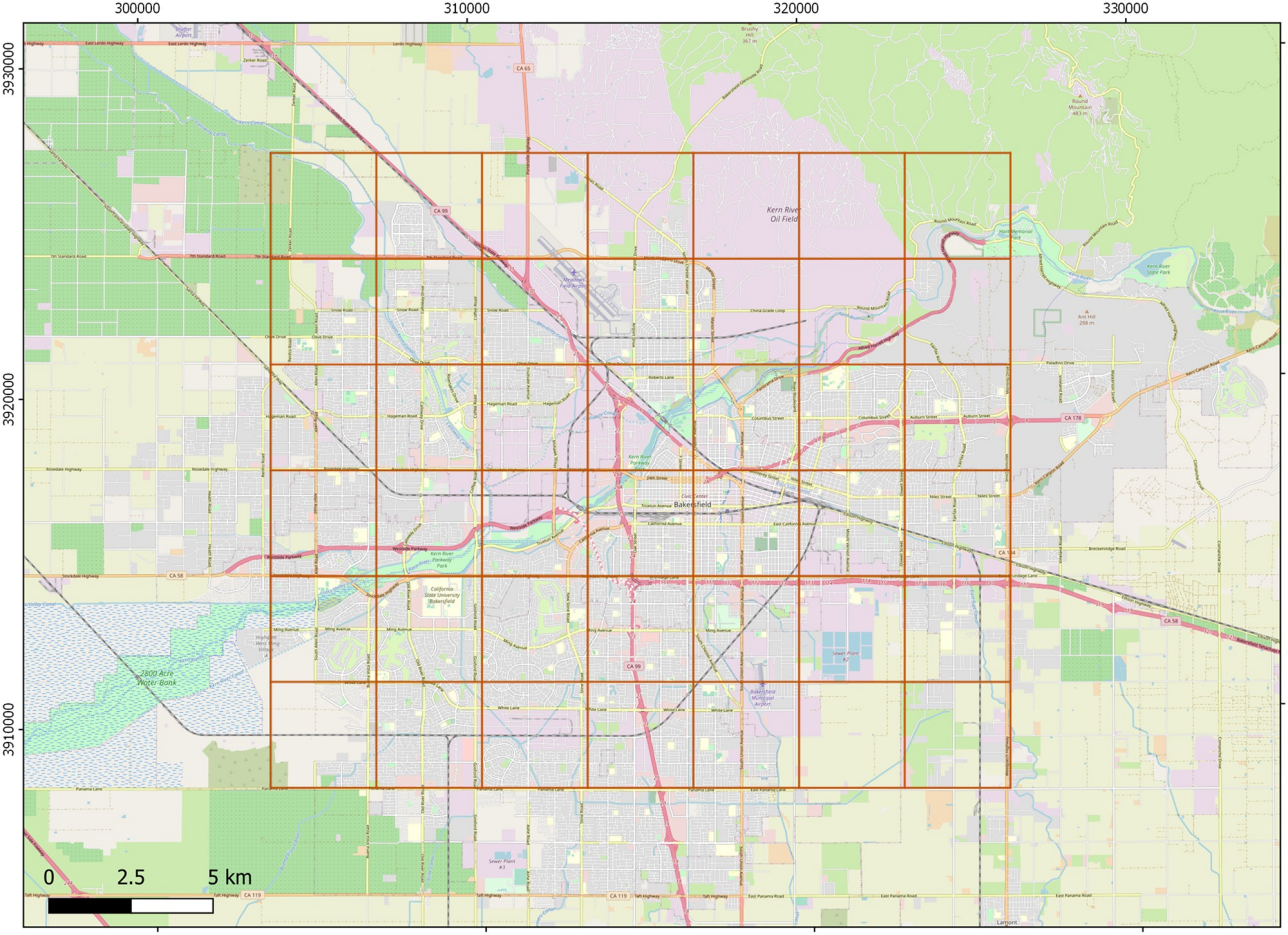
Image of Bakersfield core area occupied by San Joaquin kit foxes, divided in an array of 49 “quads”, each with its own local carrying capacity. Each grid is 2 mi^2^. Base map and data from OpenStreetMap and OpenStreetMap Foundation.

A structured metapopulation was implemented by dividing up the core area of Bakersfield into a 7x7 grid of 4 mi^2^ quads (each 2 mi x 2 mi), as shown in Fig 1. We assigned carrying capacities (in adult females) to quads in two different ways: the “flat” K landscape assigned K = 4 to each quad. The more realistic heterogeneous landscape assigned K values between 0–10 to each quad based on a map giving approximate kit fox densities in Bakersfield (Table 1) [41]. The function **seirshell** constructs a migration matrix for among-quad movement and a transmission matrix for among-quad disease transmission. There are two parameters for migration, m and α; m is the overall daily emigration rate from a quad (which is seasonally adjusted), and α governs the rate of exponential decay of the distance effect on migration. Within-quad transmission, β, is as given in the compartment model above. The parameter alphabeta (α_β_) governs the exponential decay of transmission over distance, and we create the concept ’projection’ which is the composite parameter β/α_β_. Most of the other parameters used by **metaseir** are delivered through four R dataframes (spreadsheet equivalents): foxseirscape49 which describes each quad spatially and its carrying capacity K; foxseirnaught49 which provides the initial population numbers N_0_, S_0_, E_0_, I_0_, and R_0_ for each quad; foxseirdem49 which gives the demographic features for each quad; and foxseirrates49 which gives the disease rates σ, γ, and ν for each quad. Demographic and SEIR rates can be set separately for patches in a general case, but for this paper they were held constant across quads. The function **autometabeta** allowed us to automate runs of **seirshell** for a diversity of β and α_β_ levels.

**Table 1:** Array of quads in Bakersfield with heterogeneous carrying capacity (K) set to reproduce natural differences in kit fox K across the city compared with a “flat” landscape in which each quad has the same K.

Quad ID	K per quad (natural)	K per quad (nonheterogeneous, flat)
1 2 3 4 5 6 7	0 1 2 1 0 0 0	4 4 4 4 4 4 4
8 9 10 11 12 13 14	4 5 6 6 9 2 0	4 4 4 4 4 4 4
15 16 17 18 19 20 21	10 4 6 3 3 10 2	4 4 4 4 4 4 4
22 23 24 25 26 27 28	3 4 6 1 1 8 3	4 4 4 4 4 4 4
29 30 31 32 33 34 35	3 8 10 2 6 6 1	4 4 4 4 4 4 4
36 37 38 39 40 41 42	4 6 4 4 10 6 1	4 4 4 4 4 4 4
43 44 45 46 47 48 49	1 3 6 8 10 1 0	4 4 4 4 4 4 4

In this study, each 4000-day (11 year) run started with an initial state, foxseirnaught49, which reflected the initial observed case distribution of 2013. The runs were not initialized with a single case of mange, but rather with the number observed in year one. We took this approach because: 1) we do not know where or when the initial case occurred, and 2) starting from a single infected individual would have produced a large fraction of runs in which the epidemic would have died out extremely early due to demographic stochasticity.

### Parameter estimation

The population size in Bakersfield was initiated at 400 San Joaquin kit foxes, i.e. 200 females (Table 2). Seasonal birth rate was calculated as ln(3.8/2 * the probability of an adult female birthing pups)/45 day window in which pups could be born, where 3.8 is mean llitter size and it is divided by 2 to estimate number of female pups born. We calculated the daily average natural death rate as -ln(total annual mortality) and dividing by 365 days.

**Table 2:** Definition and estimates of kit fox population variables and parameters for model metaseir, a stochastic, discrete-time compartment and metapopulation model for mange and population dynamics, applied to San Joaquin kit foxes in Bakersfield, California.

Parameter or variable	Definition	Estimate	Justification
N	Female host population size	N_0_ (starting N) = 200/Bakersfield	[21]
b_m_	Maximum birth rate at low population density OR	0.003576 (annual)	[21]
		OR	
	seasonal birth rate	0.029 (seasonal)	
Adult mortality rate	baseline mortality rate without disease	-ln(0.5)/365 = 0.001899	[42, 43]
K	Equilibrium population size due to birth and death K_b_(1-((d+d)/b_m_)	200/Bakersfield or 4/quad	[44]
m	Daily probability of individual migrating during migration period	0.00003	[21, 28–31]
α	Expected decay rate per meter of migration over distance	1/1000	[21, 30]
v_r_	Variance of r(t), the diffusion component, or environmental stochasticity	0	Could be applied if indicated in future

Epidemiological parameters were also estimated from our data, other available data for the species, and where necessary, the most closely related species for which data were available (Table 3). We assumed that once exposed, kit foxes entered and then exited the E state such that the mean incubation time is 1⁄σ. Values of α_β_ were informed by observations of dispersal distance described in the section “Kit fox movement”, i.e. 1/α_β_ ~ mean dispersal distance. The reciprocal of α_β_ (i.e. α_β_^-1^) gives the mean transmission distance, since the decay over distance is assumed exponential. Thus α_β_ of 0.6 corresponds to a mean transmission distance of 1.67 kilometers, the distance from the center to the edge of one of our patches. The disease projection β/α_β_ is a transmission measure that takes into account local transmission and the geographic impact beyond local transmission. Values of β were derived: once there was an estimate of α_β_, we calculated the value of β that produced output models that fit the observed epidemic history. Of note, concurrent to this study and data collection, we had applied a long-acting acaricide (flumethrin) to 17 kit foxes, which may have acted as a temporary form of “immunity”, essentially putting these kit foxes into an R class in the SEIR model [45].

**Table 3:** Definition and estimates of mange variables and parameters for model metaseir, a stochastic, discrete-time compartment and metapopulation model for mange and population dynamics, applied to San Joaquin kit foxes in Bakersfield, California.

Parameter or variable	Definition	Estimate	Justification
S	Number of susceptible kit fox females	Initialized as N– 9 (# initial cases)	[12]
E	Number of latently infected kit fox females	Initialized as 0	
I	Number of infectious kit fox females	Initialized as original # of cases	[12]
R	Number of recovered, immune kit fox females	0	[5, 38]
β	Transmission rate	N/A	estimated when composite β/α_β_ yields best fit to epidemic history
γ	Recovery rate	0	[5, 38]
ν	Rate of loss of immunity	N/A	[5, 38]
d_E_	Mortality rate for exposed	assume d_E_ = d_S_	
d_I_	Mortality rate for infectives	d_I_ = d_S_ + δ	
d_R_	Mortality rate for recovereds	N/A	
σ	Average incubation period, 1/σ (day^-1^)	0.047	1/21 days (3 week incubation)
α_β_	Exponential decay rate of transmission over space	N/A	1/α_β_ ~ mean dispersal distance
Ƙ	Treatment rate	0 but could be applied in future	

### Analysis

From the simulated epidemic histories we gathered and analysed the following outputs: mean epidemic time, mean kit fox population persistence time, maximum and median number of E + I, and minimum and median N (adult female kit fox population size). Local extinction of kit foxes and disease occurs when N = 0 and E + I = 0, respectively. After confirming normality, we used paired t-tests to compare mean epidemic times that occurred using the actual, heterogeneous landscape versus a flat landscape in which K was constant across all quads. Given that an influx of juvenile S individuals could impact epidemic duration and magnitude, we also examined model outputs with varying birthrates. Because mange transmission among foxes has been reported to be frequency-dependent [39], we also compared model output for frequency (by dividing the transmission function by N) and DDT. Default parameters remained static across model runs except where noted to implement the analysis; each analysis was done by running the model for at least 10 trials and 4000 days.

## Results

### Disease spatial spread

**Fig 2:**
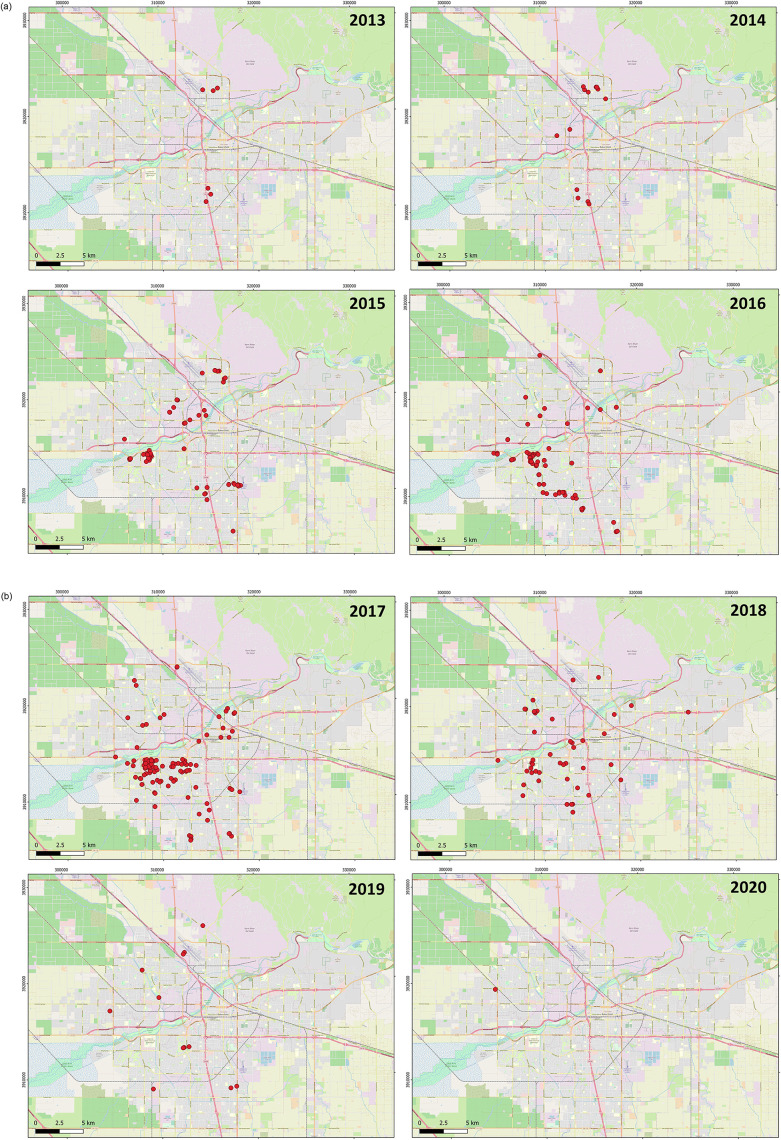
Annual locations of sarcoptic mange cases during an epidemic among San Joaquin kit foxes in Bakersfield, California from 2013–2020. Base map and data from OpenStreetMap and OpenStreetMap Foundation.

The mange epidemic was first detected on March 13, 2013 with reports of kit foxes with mange at the Meadows Field airport in the north of Bakersfield [12]. Over all of 2013, there were nine cases: three total cases at Meadows Field (through May), followed by a single case at an apartment complex just to the east in July, a case at an elementary school in central Bakersfield in late July, nearby cases at a second apartment complex in September, and then cases at a high school in northern Bakersfield in November (Fig 2; in some circumstances, “cases” represented the same fox found with a repeated case of mange). The cluster (i.e. a non-random overly dense configuration) and in the north covered approximately 1500 m in a line from west to east, while the cluster in the south extended from north to south for 1300 m. The two clusters were separated by 10,220 m.

In 2014, there were 14 cases detected. In the first quarter of the year, these included three kit foxes near Meadows Field, one to the south, and one at a new relatively industrial site called Landco approximately 5 km southwest of Meadows Field. Additional cases appeared in the third quarter in the south (three kit foxes), Landco (one), and Meadows Field (four); and in the fourth quarter, an infested kit fox was found at Oildale, 1.5 km from Meadows Field. In 2015, case numbers increased dramatically to a total of 96 cases. These included 17 cases at Landco, eight at Meadows Field, 10 at the established southern cluster, and numerous new clusters across the city including 40 cases on and near the California State University at Bakersfield (CSUB) campus.

The year 2016 saw 95 cases, including continued cases at many of the clusters, often spreading over considerably larger areas than in 2015 with at least two new clusters as well. There were 180 cases in 2017 all over the city, 52 in 2018, 29 in 2019, and only three in 2020 (although observation bias may impact case report numbers, especially during the COVID-19 pandemic in 2020). As numbers diminished in 2018, clusters began to disappear although there was a case outside town 9 km to the east of downtown. Cases appeared in Taft, southwest of Bakersfield, for the first time in 2019. Of the 2020 cases, two were in Taft and the third was northwest of the Landco cluster.

Overall, there were ten instances where a discernable, new cluster could be identified and the rate of possible spread calculated from a patch from the year before. Distances among centers of these patches ranged from 1953 m to 6836 m, and months between cases ranged from 8.5 to 19.5. This resulted in diffusion rates from 130.2 to 804.2 m/month, for an overall average of 387.2 m/month (189.1 standard deviation). During the entire Bakersfield mange epidemic between 2013–2020, the population of kit foxes dropped to about by at least half of its prior size, well below the carrying capacity set by food and shelter requirements. The epidemic in this intense form, with this level of host population reduction, persisted for six years before sharply declining.

### Individual kit fox movement

**Fig 3:**
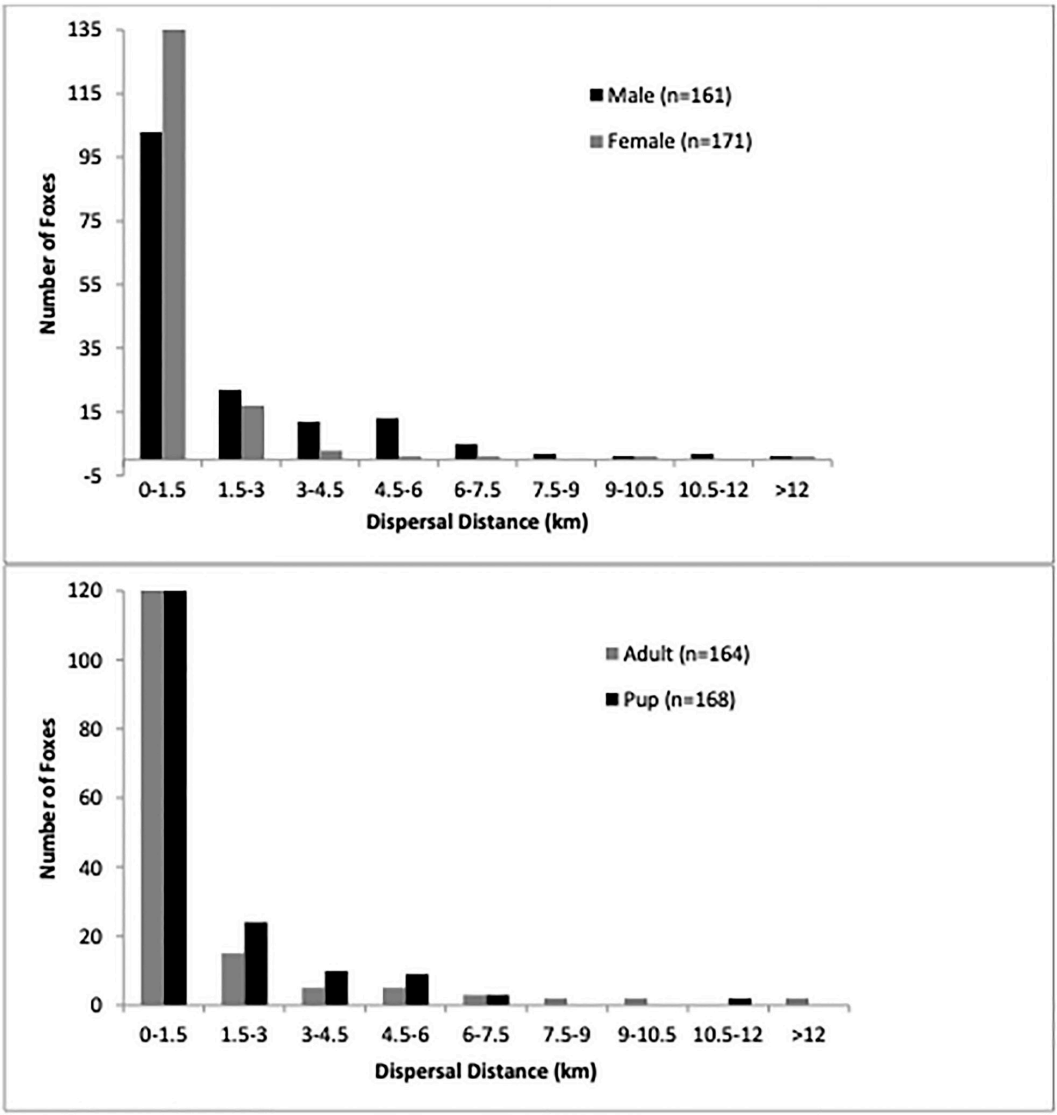
Summary of distances moved by San Joaquin kit foxes in Bakersfield, California, inferred from re-trapping or found carcasses.

Among 332 kit foxes for which recapture or telemetry data allowed measurement of maximum known linear distance moved, the apparent pattern was exponential decay with a mean distance moved of 1.08 km. Most (75.3%) tended to remain very close to their capture site moving less than 1.5 km (Fig 3). Among longer-distance travelers (> 3.0 km), the mean distance was 5.70 km, and the overall maximum was 12.79 km. The tendency to remain within 3.0 km of the capture site was considerably stronger among females (95.9% of all females), who moved on average 0.99 km compared with 1.17 km in males (77.6% remaining within 3.0 km); this difference was statistically significant (p = 4.4 x 10^−7^). On average pups < 1 year old moved 1.12 km and 85.7% remained within 3.0 km compared with 1.03 km mean distance among adults, of which 88.4% remained within 3.0 km. Longer distance movement probability did not differ significantly between age groups (p = 0.52). Fourteen kit foxes moved > 6.0 km. Of these 14, 11 were male and three female, and nine were adults and five pups.

### Fitting the model to the epidemic

**Fig 4:**
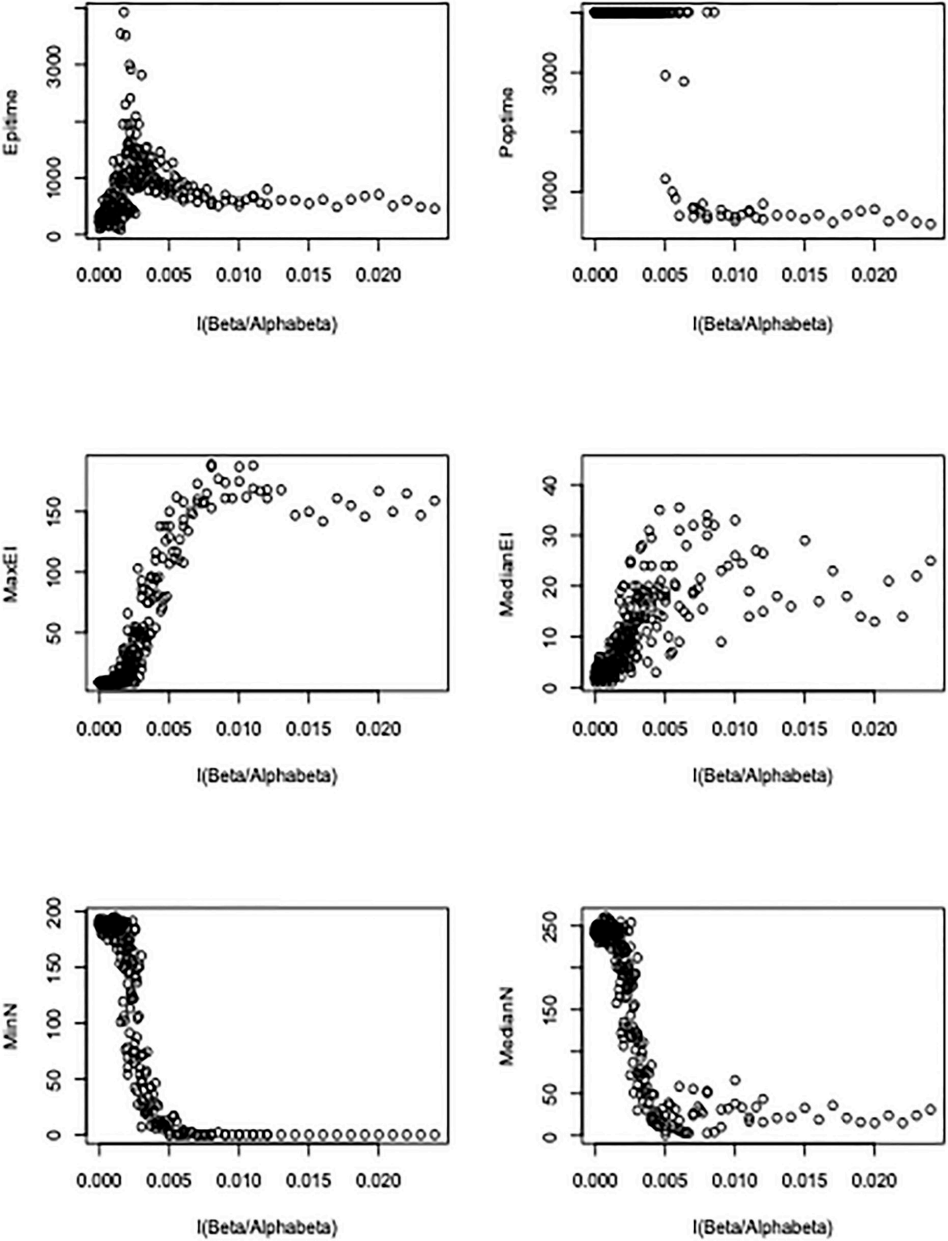
The effect of disease projection (β/α_β_) on metaseir-simulated epidemic times (Epitime), kit fox population persistence time (Poptime), number of infected individuals (MaxEI, MedianEI), and kit fox population size (MinN, MedianN). Data points were generated by the call: > h = 49; beta49.3noEIbirth = autometabeta(trials = 1, betav = seq(0.0000,0.0024,0.0001), alphabetav = seq(0,1,0.1)). This runs **metaseir** 275 times, covering a variety of beta and alphabeta values.

During exploration of the model space, it became apparent that the composite parameter β/α_β_ closely predicts epidemic times, numbers in the E and I compartments, and kit fox population size (Fig 4). Because β measures local disease transmission and α_β_ measures the decay of transmission over distance, then 1/α_β_ is the mean distance of transmission, and β/α_β_ measures, in some sense the **projection** of the disease across space.

**Fig 5:**
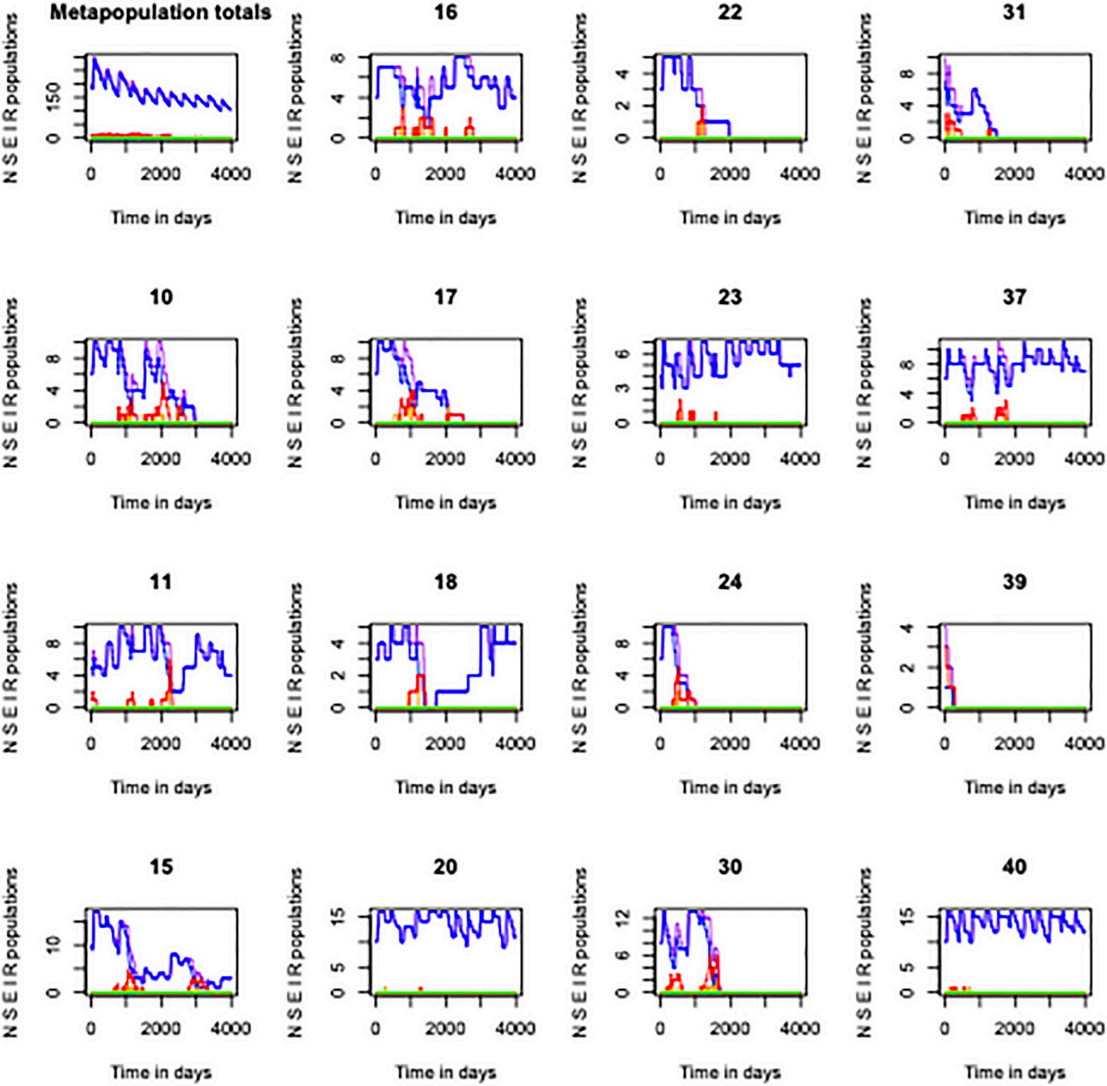
Examples of runs of simulation metaseir, which is called by seirshell. The figure plots overall metapopulation numbers and outcomes in 15 patches (excluding patches where local K = 0 and including diversity among the remaining patches with respect to K) to illustrate the types of results that can be obtained. N, S, E, I, R numbers are shown in purple, blue, orange, red, green respectively. This simulation run with β = 0.0015, a_b_ = 0.6, m = 0.00005 and α = 0.001 led to an epidemic persistence time of 3350 days (9.2 years), and the kit fox population dropped to about half of its starting point. Both kit fox and parasite populations show metapopulation dynamics with local extinction and colonization, with the annual birth pulse evident in the metapopulation totals panel. Data were generated in R by the function call: > h = 49; fox49outmarch2 = seirshell(trials = 1,tmax = 4000,h = 49, beta = 0.0015, alphabeta = 0.6, EIbirth = F,BetaHwander = diag(0,h,h), seirscape = foxscape49, seirdem = foxseirdem49, seirrates = foxseirrates49, seirnaught = foxseirnaught49); animetaseir(fox49outmarch2$meanmfx,4,4, patchdisplay = c(10,11,15,16,17,18,20,22,23,24,30,31,37,39,40),deathtrace = F);fox49outmarch2$epitimes.

Our simulation **metaseir** produced epidemic histories which were consistent with empirical epidemic observations when run with a projection value of approximately β/α_β_ = 0.0025. Exploration of model output across a range of β/α_β_ revealed relevant regions of parameter space. When β/α_β_ > 0.0050, a fierce epidemic lasted about two years, during which time the kit fox population went effectively extinct. When β/α_β_ ≈ 0.0025, it was possible for the epidemic to last the maximum amount of time for this model with these parameters, about six years. Of note, only a fraction of runs yielded epidemics of 6 years, with mean epidemic time often closer to four years. At this level of transmission, the kit fox population declined by about half. As β/α_β_ was reduced below 0.0025, the disease typically died out quickly without impacting the host population. Fig 5 gives an example of typical kit fox/mange dynamics when the β/α_β_ = 0.0015/0.6 = 0.0025, showing good fit to the observed Bakersfield mange epidemic as well as kit fox and parasite metapopulation dynamics with local extinction and colonization. Note that the epidemics are longest when the projection value is approximately 0.0025 for a broad range of β, but at highest levels of β, the projection yielding long epidemics was 0.003.

Because the best simulation fit to the actual Bakersfield mange epidemic occurs when ^®^/α_β_ ≈ 0.003, we can calculate approximate β and α_β_ estimates if we make the assumption that the mean disease transmission distance should be approximately the same as the mean movement distance, which is 1.08 km. Thus we estimate α_β_ = 1/1.08 km = 0.926 km^-1^ and a plausible fit for the local transmission rate β is β = 0.0025*0.926 = 0.00213. A slightly better fit is obtained by using the optimal projection value (0.003) for higher β.

When α_β_ values are close to zero (i.e. no transmission decay over space) and ^®^/α_β_ is close to 0.0025, we can still get epidemic times that are high, which would imply that spatial structure is not critical for the maintenance of the disease. However, given the β value calculated above, a value of α_β_ near zero would lead to rapid extinction of the kit fox population and the epidemic.

As parameterized, the model allowed for natural deaths all year, augmented with death due to mange. In contrast, births occurred only in a 45 day window, during which births would have to compensate for death for the population to persist. When b was very low (0.0081 births/day), the host population was not maintained regardless of disease, with exponential decline in N and shorter epidemic durations. Above b = 0.0081 and using the optimized value of β/α_β_, the kit fox population was relatively insensitive to b. We did not see any part of the space when disease went extinct before the host did so. We also ran **metaseir** comparing FDT and DDT. The overall curves produced for hundreds of runs, varying β crossed with diverse values α_β_, were very similar to those of Fig 5. In particular, the epidemic time curve and the host population persistence curve also showed a critical dependence on β/α_β_. Examples are provided in the S1 Appendix. Of note, in order for the epidemic to persist under FDT, β/α_β_ had to be an order of magnitude higher than for DDT.

Lastly, we compared simulation output between the flat and heterogeneous landscapes. Epidemic times were slightly longer for the heterogeneous landscape when β/α_β_ is below 0.003, and slightly longer for the flat landscape above 0.003. The main landscape effect we observed is this shift in the projection value yielding the longest epidemics, i.e. stronger projection is needed in the flat landscape to get a long epidemic.

## Discussion

Epidemics of sarcoptic mange, or any highly contagious, fatal disease for which hosts have little to no immunity, may be characterized by rapid-onset, high amplitude, intense regulation of host populations, and then extinction of the disease [46]; in other cases, the epidemics tend towards endemicity. Thus it is enigmatic that mange could suppress a small isolated population of endangered kit foxes by at least half and yet both kit foxes and mange persist for almost a decade without going extinct. These dynamics suggest that mange is regulating the San Joaquin kit fox population in the sense discussed by Hassell et al. [47]. Neither the existence of infested dens [16, 48] nor reservoir hosts [17] satisfactorily predicts patterns of mange persistence observed in San Joaquin kit foxes. In contrast, sharing of mange among kit fox family groups in spatially semi-discrete patches can better account for a mange epidemic with rapid onset, suppression of the host population at about 50% of pre-mange levels, and years-long persistence.

In contrast with literature suggesting that mange has FDT [38], we did not find that the mode impacted the outcome of the metapopulation modeling runs other than to require that the projection would need to be much higher than for DDT for an epidemic to persist at all. We also note that some researchers have found that rates of mange were positively correlated with host density, suggesting that FDT is not a universal attribute of mange [1, 8, 49].^.^ A likely explanation for our findings is that there are of course two spatial scales at which mange transmission matters in this kit fox epidemic: local families of kit foxes interacting within a patch, and across-patch transmission during kit fox movement. Each patch features such a small number of kit foxes that stochasticity heavily influences disease transmission, time of death, and kit fox demography, “drowning out” the signal of possible influences of FDT. FDT within a patch is plausible if each kit fox tends to seek out a set number of other kit foxes (although natural history data as described above indicate that non-family interactions are rare). In contrast, among patches, the likelihood of interacting with other kit foxes seems more likely to be a function of population density. Admittedly, overall metapopulation density and colonization among patches could be influences by local patch densities, with high density in a patch tending to encourage an individual to forage over greater distances or emigrate. Such scenarios could be further explored both *in silico* and by observations in the field.

During the epidemic, our team was also engaged in trapping or accepting very ill kit foxes from the public and working to treat and re-release them when possible [45]. We acknowledge that these interventions could have changed population and disease dynamics: treating and re-releasing recovered foxes could have prolonged the slow simmer of the epidemic while our modest deployment of long-acting acaricides could have briefly and locally reduced force of infection. A considerable benefit to **metaseir** is to serve as a population viability analysis (PVA) tool, given its capacity to incorporate such interventions and make predictions as to their impact, improving management options.

In the face of attempted treatment and the ongoing epidemic, our main findings from **metaseir** model were that: 1) a simple metapopulation model can capture the Bakersfield-like disease epidemic dynamics even without any environmental reservoir or chronically-infected alternative host, 2) the parameter that has the most effect on persistence and magnitude of the epidemic is the projection, β/α_β_, in the range of 0.0025–0.003, 3) carrying capacity heterogeneity shifts the critical projection value downwards, i.e. if foxes were more evenly distributed, higher β/α_β_ values would be needed to get persistent epidemics, and 4) the disease epidemic is relatively insensitive to birth rates.

Both kit foxes and mange are in a metapopulation and thus, in addition to the built-in landscape heterogeneity, historical contingency yields heterogeneity over time and across quads in kit fox numbers and mange cases (which Keeling calls dynamic heterogeneity [50]). Other features of the landscape may contribute to heterogeneity, such as large roads where kit foxes may be killed by traffic or the Kern River, which can be a barrier to movement or its adjacent riparian vegetation which serves as a corridor for movement. We acknowledge that case data may not be complete because case reports were a combination of passive and sporadic but localized intensive prospective surveillance. We have previously suggested that case data are robust because day-active San Joaquin kit foxes are highly visible and in many cases beloved by local residents, occupying habitat such as people’s porches, school athletic fields, and other locations where presence of a dead kit fox or sudden absence of a regularly observed kit fox family would not be overlooked [13].

Our estimate of disease diffusion rate is acknowledgedly imprecise as well. We used median month of detection of any new patch rather than earliest to allow for more data (cases) to be included in any new patch, but this may result in an estimate which appears a little slower than had we used earliest. However, given how wide a range of values there was for rate of establishment of new patches, imprecision in median vs. initial date may have had little impact. Diffusion was studied in chamois in northern Italy as well (called “oil-drops” by the authors) averaging between 2.4 and 4.5 km/year with some saltation as well [51], while a more recent study also found about 4.5 km/year spread [52]. Spread of mange among wombats was described as via a travelling wave with variable year to year speed [16]. Our data on kit fox movement likely omitted some animals with very long distance dispersal, and research on sarcoptic mange in wolves *(Canis* lupus) experienced similar difficulties [53]. Lastly, there is variance around all parameters and outcomes. Although **metaseir** was able to capture the observed epidemic dynamics, many modeling runs terminated the epidemic much faster than 7–8 years. For example, with β = 0.0004 and β/α_β_ = 0.002, only 28% of the simulated epidemics lasted longer than 2000 days. With β = 0.0012 and β/α_β_ = 0.002, 20% of the simulated epidemics lasted longer than 2000 days. These data imply that the epidemic which did occur may have been unusually severe relative to average predictions.

Despite biases however, our data on kit fox movement and spatial distribution of mange cases both support the plausibility of spatial heterogeneity in contact among patches. *A priori* we would expect heterogeneity to promote longer disease persistence and the heterogeneous landscape experienced a slight but significantly increased epidemic. However, for this species, each grid has a very low N, such that stochasticity and high variance in demographic features play an important role in epidemic and population trajectories, quads can go extinct, and they are not quickly replaced. This context could help explain why the model was not particularly sensitive to changes in birth rate: as long as births exceed deaths, each quad can come to its carrying capacity and in doing so, refuel the S compartment with susceptible individuals. Our flumethrin treatment could also impact epidemic trajectory. While it was deemed necessary for the health of individual kit foxes, it did not appear to dampen the overall epidemic and indeed, as kit foxes lost their artificial immunity (i.e. the immunity conferred by the collars), they also re-entered the S class and could have fueled spread.

**metaseir** was intentionally written to be generalizeable for use with other situations, for example by including recovery and immunity even though those were set to 0 for the mange simulation. Importantly, the model could guide expectations during an intervention using long-acting acaricides which afford individual kit foxes months of protection [45] and could function as a form of herd immunity; this could be modeled easily by making the recovery rate k non-zero and allowing some treated kit foxes to reside in the R (recovered) compartment transiently. Notably, having treated and untreated foxes would further contribute to population heterogeneity. While not proposed for this species, the model can also be used to assess impacts of culling on likelihood of epidemics occurring and magnitude and duration of any ensuing epidemics. For the endangered San Joaquin kit fox, use of the model may be crucial to explore how the epidemic may ensue if it spreads to exurban and larger subpopulations, particularly given the few cases already observed in Taft. However in any exurban epidemic, it will be important to update parameters, particularly because the local carrying capacity would be lower and movement distances could be greater. These would yield a different value of the key projection composite.

When applied to mange in other species, our results here suggest that the focus in data collection should be less on host demography and more on whether observed epidemics are transient, whether the hosts have variance in contact rates and transmission, and whether their K landscape is “flat” or heterogeneous. For example, red foxes experience extremely severe mange associated with potential local extinction [4], which could plausibly be due to particularly high β or β/α_β_. In other early mange epidemics, the impact can be as considerable as a reduction in 98% in Spanish ibex and 80% in chamois [6, 51]. Over time, populations may rebound to 67–75% of the original [51]. Further exploration of how mange differs in communities with multiple vs. single affected species, with and without FDT, and with and without immunity could feature use of the model guided by field data.

Our data can be used to estimate R_0_. The early-epidemic annual disease multiplier can be calculated as 10.7 because there were seven known cases in 2014 and 75 in 2015. Because R_0_ is disease multiplier for one transmission generation, we need to know the disease transmission generation time. In a hypothetical, extreme case, a kit fox incubates (mean time in state E) for 21 days, lives for 100 days (mean time in state I), but only transmits for a few days towards the end of her life. In this case the T_gen_, the transmission generation time, is 121 days or three generations/, and thus R_0_ is 10.7 ^(1/3) = 2.2. Alternatively, if the kit fox transmits through the I state, then its T_gen_ = 21 + 100/2 = 71 days. With five generations of mange per year, R_0_ = 10.7 ^(1/5) = 1.6. Other studies approximated the R_0_ of mange in red foxes as 2.67 [38], and in chamois as 4.8–5.1 [54]. Our annual multiplier 10.7 is likely to underestimate R_0_ because we have assumed exponential growth in I which is only plausible until the available S pool begins to decline. At this point, cases are multiplying according to a new “effective multiplier” (R_e_), a non-static statistic similar [55] which highlights that the number of new cases is expected to change as an epidemic progresses.

Despite being a common disease, our fundamental understanding of mange epidemiology in wildlife is poor [49, 56]. Epidemics of sarcoptic mange often feature an initial peak with high numbers of infested and dying individuals, after which mange often becomes endemic. In some cases, there appear to be epidemic waves approximately every 10–15 years [1, 8]. A tool like **metaseir** can be used to efficiently explore dynamics to better reveal drivers that plausibly explain patterns observed across wildlife species, such as cyclic epidemic waves, endemicity or disease extinction, and disease-imposed regulation of host population sizes. With remaining San Joaquin kit foxes numbering less than 5,000 across their metapopulation [22, 23], every remaining subpopulation is critically important for conservation. As one of the largest remaining, the until-recently stable subpopulation in Bakersfield [21, 57] is crucial for conservation and recovery as a hedge against catastrophic events in natural lands, a source of genetic diversity, and a potential source for reintroductions [21, 58]. Integration of disease and host dynamics into a metapopulation model provides a valuable tool to improve efficiency of management for iconic Bakersfield and exurban San Joaquin kit foxes. Systematic exploration of epidemiological data and synthetic modeling can also advance our understanding of the ecologies of high amplitude yet persistent epidemics of other highly lethal diseases.

## Supporting information

S1 Appendix Characteristics of mange epidemics when beta is frequency-dependent. (DOCX)
