# Supplementary material for: Spatio-temporal and transmission dynamics of sarcoptic mange in an endangered New World kit fox
Source: PLoS One. 2023 Feb 16;18(2):e0280283. doi: 10.1371/journal.pone.0280283 (PMC9934372; doi:10.1371/journal.pone.0280283)
Supplement: S1 Appendix — (DOCX) [file pone.0280283.s001.docx]

**Appendix.** Characteristics of mange epidemics when beta is frequency-dependent.

beta epi-duration poptimes maxEI

1 0.01 237.9 2010.0 2.86

2 0.02 715.0 1995.6 15.46

3 0.03 718.0 1743.6 31.25

4 0.04 856.0 1565.0 50.29

5 0.05 790.0 1374.9 58.30

6 0.06 799.0 1303.0 66.73

7 0.07 805.1 1210.5 77.30

8 0.08 762.7 1244.1 82.78

9 0.09 791.9 1244.6 92.85

10 0.10 722.0 1070.4 99.54

11 0.12 763.8 1000.8 116.60

12 0.14 716.1 1010.5 119.50

13 0.16 761.7 990.6 130.40

14 0.18 759.1 864.1 142.50

15 0.20 696.8 862.4 144.60

16 0.22 719.5 879.2 144.21

17 0.24 700.8 876.4 148.69

18 0.26 742.8 789.6 159.77

19 0.28 762.9 804.0 162.35

20 0.30 729.0 760.0 165.00

21 0.32 722.6 802.3 160.00

22 0.34 671.2 753.6 160.65

23 0.36 732.9 828.6 162.01

24 0.40 780.6 784.6 168.94
